# The E3 ubiquitin ligase TRIM31 attenuates NLRP3 inflammasome activation by promoting proteasomal degradation of NLRP3

**DOI:** 10.1038/ncomms13727

**Published:** 2016-12-08

**Authors:** Hui Song, Bingyu Liu, Wanwan Huai, Zhongxia Yu, Wenwen Wang, Jing Zhao, Lihui Han, Guosheng Jiang, Lining Zhang, Chengjiang Gao, Wei Zhao

**Affiliations:** 1 Department of Immunology, Shandong University School of Medicine, Jinan, Shandong 250012, China; 2 Key Laboratory of Infection and Immunity of Shandong Province, Shandong University School of Medicine, Jinan, Shandong 250012, China; 3 Institute of Basic Medicine, Shandong Academy of Medical Sciences, Jingshi Road 18877, Jinan, Shandong 250062, China

## Abstract

The NLRP3 inflammasome has a fundamental role in host defence against microbial pathogens and its deregulation may cause diverse inflammatory diseases. NLRP3 protein expression is a rate-limiting step for inflammasome activation, thus its expression must be tightly controlled to maintain immune homeostasis and avoid detrimental effects. However, how NLRP3 expression is regulated remains largely unknown. In this study, we identify E3 ubiquitin ligase TRIM31 as a feedback suppressor of NLRP3 inflammasome. TRIM31 directly binds to NLRP3, promotes K48-linked polyubiquitination and proteasomal degradation of NLRP3. Consequently, TRIM31 deficiency enhances NLRP3 inflammasome activation and aggravates alum-induced peritonitis *in vivo*. Furthermore, TRIM31 deficiency attenuates the severity of dextran sodium sulfate (DSS)-induced colitis, an inflammatory bowel diseases model in which NLRP3 possesses protective roles. Thus, our research describes a mechanism by which TRIM31 limits NLRP3 inflammasome activity under physiological conditions and suggests TRIM31 as a potential therapeutic target for the intervention of NLRP3 inflammasome related diseases.

## Abstract (web summary)

The NLRP3 inflammasome controls the response of the host to pathogens; precise regulation is required to limit autoimmune diseases. Here, the authors identify the E3 ligase TRIM31 that aids the ubiquitin-mediated proteasomal degradation of NLRP3, which may be a therapeutic target for alleviating disease.

NLRP3 inflammasome is a multi-protein platform which comprises NLRP3, ASC and caspase-1, and plays crucial roles in host defence against pathogens^1^,^2^,^3^,^4^,^5^. Inflammasome complex assembly is triggered by stimuli from both microbial infection and endogenous ‘danger signal', such as nigericin, crystals, extracellular ATP, amyloid-β and alum and so on. And then the inflammasome complex serves as platforms for the activation of the cysteine protease caspase-1, which cleaved pro-IL-1β and pro-IL-18 into mature IL-1β and IL-18 (refs 1, 2, 3, 4, 5). NLRP3 inflammasome has been implicated in many kinds of diseases, such as gout, autoimmune disorders, atherosclerosis, type 2 diabetes and obesity^5^,^6^,^7^,^8^,^9^. Thus, NLRP3 inflammasome activity must be tightly controlled to maintain immune homeostasis and avoid detrimental effects.

NLRP3 expression level is considered as a limiting step in inflammasome activation^10^,^11^. In resting macrophages, the protein level of NLRP3 is relatively low, so that NLRP3 inflammasome assembly is hardly induced^11^,^12^. Induction of NLRP3 protein expression licensed by TLR ligands (signal 1) allows respective NLRP3 activators (signal 2) to trigger caspase-1 cleavage^10^,^13^. Thus, regulation of NLRP3 level offers an interesting mechanism to alter the inflammatory potential of immune cells^11^. Up to now, several mechanisms for negative regulation of NLRP3 expression have been established. For example, we reported that aryl hydrocarbon receptor could bind to the NLRP3 promoter and inhibit its expression at transcriptional level^14^. MiR-223 suppresses NLRP3 mRNA expression through a conserved binding site within the 3′ untranslated region of NLRP3 (refs 15, 16).

Autophagy–lysosomal pathway and ubiquitin–proteasome pathway are two major protein degradation systems widely exist in mammalian cells, which provide specificity and regulate the intensity of innate immune responses. It has been reported that autophagy-dependent degradation was involved in the regulation of NLRP3 expression^17^,^18^. For example, plasminogen activator inhibitor type 2 enhances NLRP3 degradation in an autophagy-dependent manner^17^. Dopamine D1 receptor (DRD1) signalling promotes NLRP3 ubiquitination via E3 ubiquitin ligase MARCH7, leading to the autophagy-mediated degradation of NLRP3 (ref. 18). However, whether ubiquitin–proteasome pathway is also involved in the regulation of NLRP3 protein expression remains unknown.

TRIM family proteins have been implicated in the negative regulation of innate immune responses by promoting degradation of their respective substrates through ubiquitin–proteasome pathway^19^. TRIM31 is a member of the TRIM protein family, which is encoded within the major histocompatibility complex (MHC) class I region^20^. Several TRIM family members (including TRIM31, TRIM40, TRIM10, TRIM15, TRIM26 and TRIM39) are organized in a tight cluster and two TRIM genes (including TRIM38 and TRIM27) telomeric of the cluster within the MHC class I region^20^. Most of the above mentioned TRIM family members possess vital regulatory effects in innate immune responses^19^,^21^,^22^,^23^,^24^,^25^,^26^,^27^. However, the biological function of TRIM31, especially in the innate immune response, remains unknown.

In this report, we describe a mechanism by which TRIM31 negatively regulates NLRP3 inflammasome activity. TRIM31 could directly bind to NLRP3, and then promote K48-linked polyubiquitination and proteasomal degradation of NLRP3 in both resting and activated macrophages. The identification of TRIM31 as a physiological suppressor of NLRP3 expression provides an explanation on the limitation of NLRP3 inflammasome activation under physiological conditions. Consistent with that, TRIM31 deficiency facilitated NLRP3 inflammasome activation, enhanced IL-1β secretion and thus aggravated alum-induced peritonitis *in vivo*. On the other hand, TRIM31 deficiency attenuated the severity of dextran sodium sulfate (DSS)-induced colitis by promoting NLRP3 inflammasome activation, which was reported to be possessing protective roles in the model. Thus, TRIM31 could be a potential therapeutic target for NLRP3-associated syndromes, including autoinflammatory and autoimmune diseases.

## Results

### TRIM31 specifically inhibits NLRP3 inflammasome activation

**Figure 1:**
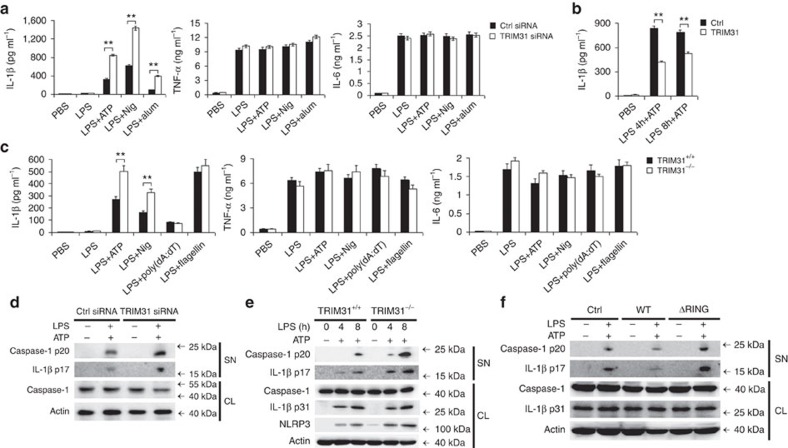
TRIM31 specifically inhibits NLRP3 inflammasome activation. (**a**) ELISA of IL-1β, TNF-α and IL-6 in supernatants from mouse peritoneal macrophages silenced of TRIM31, primed with LPS for 8 h, and followed by stimulation with ATP, Nig. or alum for 30 min. (**b**) ELISA of IL-1β in supernatants from THP-1 cells transfected with TRIM31 plasmid, primed with LPS for various times, and followed by stimulation with ATP for 30 min. (**c**) ELISA of IL-1β, TNF-α and IL-6 in supernatants of mouse peritoneal macrophages from TRIM31^+/+^ or TRIM31^−/−^ mice, primed with LPS for 8 h, and followed by stimulation with ATP, Nig., poly(dA:dT) or flagellin for 30 min. (**d**) Immunoblot analysis of supernatants (SN) or cell lysates (CL) from mouse peritoneal macrophages silenced of TRIM31, primed with LPS, and followed by stimulation with ATP for 30 min. (**e**) Immunoblot analysis of supernatants (SN) or cell lysates (CL) of mouse peritoneal macrophages from TRIM31^+/+^ or TRIM31^−/−^ mice, primed with LPS, and followed by stimulation with ATP for 30 min. (**f**) Immunoblot analysis of supernatants (SN) or cell lysates (CL) from THP-1 transfected with TRIM31 WT or ΔRING mutant, primed with LPS, and followed by stimulation with ATP for 30 min. Similar results were obtained in three independent experiments. **P<0.01. (Student's *t*-test). Data are representative of three experiments (mean and s.d. of six samples in **a**–**c**).

To investigate the potential functions of TRIM31 in innate immune response, specific and effective siRNA targeting TRIM31 was used to suppress the expression of endogenous TRIM31 in mouse macrophages (Supplementary Fig. 1a). TRIM31 knockdown has no regulatory effects on mRNA expression of lipopolysaccharide (LPS)-induced proinflammatory cytokines, including TNF-α and IL-1β (Supplementary Fig. 1b). But, IL-1β secretion was significantly increased in TRIM31 silenced macrophages primed by LPS and then treated by NLRP3 inflammasome activator such as ATP, nigericin or alum (Fig. 1a). However, TNF-α and IL-6 secretion were not influenced by TRIM31 knockdown (Fig. 1a). Similarly, TRIM31 overexpression greatly inhibited IL-1β secretion in human THP-1 cells (Fig. 1b). To confirm the inhibitory role of TRIM31 in IL-1β secretion, the effects of TRIM31 deficiency on IL-1β expression in mouse peritoneal macrophages were observed. ATP and Nig stimulated IL-1β secretion by LPS-primed TRIM31-deficient macrophages was significantly increased (Fig. 1c, Supplementary Fig. 1c–e). However, IL-1β secretion stimulated by poly(dA:dT) (an AIM2 inflammasome activator) and flagellin(a NLRC4 inflammasome activator) was not influenced by TRIM31 deficiency (Fig. 1c, Supplementary Fig. 1d,e). In addition, TRIM31 deficiency also enhanced ATP stimulated IL-1β secretion in PGN-primed macrophages (Supplementary Fig. 1f). Furthermore, TRIM31 deficiency also had no inhibitory effects on TNF-α, IL-6 expression and IL-1β mRNA expression (Supplementary Fig. 1g). Taken together, these data indicated that TRIM31 specifically inhibited NLRP3 inflammasome activation.

Caspase-1 cleavage is a critical step for the NLRP3 inflammasome activation. We then investigated the effects of TRIM31 on caspase-1 cleavage. TRIM31 knockdown and deficiency both significantly enhanced caspase-1 cleavage in LPS-primed mouse peritoneal macrophages treated by ATP (Fig. 1d,e). Accordingly, less cleaved caspase-1 was detected in TRIM31 overexpressed THP-1 cells (Fig. 1f). However, the TRIM31 truncated mutant ΔRING, in which the N-terminal RING domain was deleted and the potential E3 ubiquitin ligase activity was deprived, lost the ability to inhibit caspase-1 cleavage, compared with TRIM31 WT (Fig. 1f).

To further rule out the possible regulatory effects of TRIM31 in the priming process of NLRP3 inflammasome activation, we explored the function of TRIM31 on NF-κB and MAPK activation. TRIM31 knockdown had no influence on LPS-induced phosphorylation of IκB-α, JNK, ERK and p38 (Supplementary Fig. 1h). Additionally, no differences of MyD88- and TNF-α-induced NF-κB reporter gene activation were observed in TRIM31 overexpressed HEK293T cells (Supplementary Fig. 1i). Collectively, these data indicated that TRIM31 specifically inhibited NLRP3 inflammasome activation and subsequent IL-1β secretion.

### TRIM31 promotes proteasomal degradation of NLRP3

**Figure 2:**
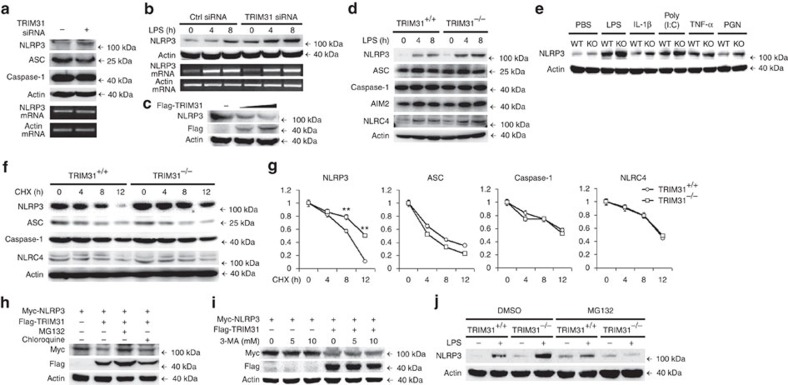
TRIM31 promotes proteasomal degradation of NLRP3. (**a**) Immunoblot analysis of extracts (upper panel) or RT-PCR analysis (lower panel) of mouse peritoneal macrophages silenced of TRIM31. (**b**) Immunoblot analysis of extracts (upper panel) or RT-PCR analysis (lower panel) of mouse peritoneal macrophages silenced of TRIM31, then stimulated for various times with LPS. (**c**) Immunoblot analysis of extracts from HEK293T cells transfected with increasing amount of TRIM31 expression plasmid. (**d**) Immunoblot analysis of extracts from TRIM31^+/+^ or TRIM31^−/−^ mouse peritoneal macrophages, then stimulated for various times with LPS. (**e**) Immunoblot analysis of NLRP3 expression from TRIM31^+/+^ or TRIM31^−/−^ mouse peritoneal macrophages, then stimulated with LPS, IL-1β, poly(I:C), TNF-α or PGN for 8 h. (**f**,**g**) Immunoblot analysis of extracts from TRIM31^+/+^ or TRIM31^−/−^ mouse peritoneal macrophages stimulated with LPS for 4 h, and then treated for various times with cycloheximide (CHX). NLRP3, ASC, Caspase-1 and NLRC4 expression levels were quantitated by measuring band intensities using ‘ImageJ' software. The values were normalized to actin (**g**). **P<0.01. (mean and s.d. of three samples in **g**, Student's *t*-test). (**h**) Immunoblot analysis of extracts from HEK293T cells transfected with Myc-NLRP3 and Flag-TRIM31 expression plasmid then treated with MG132 (10 μM) or chloroquine (10 μM) for 4 h. (**i**) Immunoblot analysis of extracts from HEK293T cells transfected with Myc-NLRP3 and Flag-TRIM31 expression plasmid then treated with 3-MA as indicated for 4 h. (**j**) Immunoblot analysis of NLRP3 expression from TRIM31^+/+^ or TRIM31^−/−^ mouse peritoneal macrophages stimulated with LPS for 4 h, together with DMSO or MG132 (10 μM) treatment for 4 h. Similar results were obtained in three independent experiments.

TRIM family proteins have been implicated in the negative regulation of innate immune responses by promoting degradation of their respective substrates through ubiquitin–proteasome pathway^19^. We next sought to determine whether TRIM31 could inhibit NLRP3 inflammasome activation by promoting protein degradation of inflammasome components. TRIM31 knockdown greatly increased NLRP3 protein expression in resting mouse peritoneal macrophages, with no influence on ASC and caspase-1 protein expression (Fig. 2a). Similarly, TRIM31 knockdown also significantly increased NLRP3 protein level in LPS-priming macrophages (Fig. 2b). But, TRIM31 knockdown had no effects on NLRP3 mRNA expression (Fig. 2a,b). Consistently, TRIM31 overexpression greatly decreased NLRP3 protein expression in a dose-dependent manner in HEK293T cells (Fig. 2c). Importantly, similar results were also observed in TRIM31 deficient mouse peritoneal macrophages. TRIM31 deficiency greatly enhanced NLRP3 protein expression in both resting and activated macrophages (Fig. 2d, Supplementary Fig. 2). But, TRIM31 deficiency could not increase AIM2 and NLRC4 expression (Fig. 2d). We next examined the effects of TRIM31 deficiency on other agonists-induced NLRP3 expression. TRIM31 deficiency greatly enhanced IL-1β-, poly(I:C)- and PGN-induced NLRP3 expression in macrophages (Fig. 2e). However, no marked difference of TNF-α-induced NLRP3 expression was observed in TRIM31 deficient macrophages (Fig. 2e).

Given the functions of TRIM family members in promoting the degradation of their target proteins, we inferred that TRIM31 might inhibit NLRP3 protein expression via promoting its degradation. TRIM31 deficiency inhibited NLRP3 protein degradation (Fig. 2f,g). However, no differences of ASC, Caspase-1 and NLRC4 protein degradation were observed (Fig. 2f,g). These data indicated that TRIM31 inhibited NLRP3 expression via promoting its protein degradation. We then explored the degradation pathway of NLRP3 mediated by TRIM31. TRIM31-induced NLRP3 degradation could be reversed by proteasome inhibitor MG-132, but not by lysosome inhibitor chloroquine (Fig. 2h) or autophagy inhibitor 3-MA (Fig. 2i), indicating that TRIM31 mediated NLRP3 degradation in proteasome. Next, we examined the NLRP3 expression in both wild type (WT) and TRIM31-deficient macrophages following MG132 treatment. MG132 treatment slightly enhanced NLRP3 expression in unactivated WT macrophages, with no effects on LPS-primed WT macrophages (Fig. 2j). In MG132-treated macrophages, TRIM31 deficiency could not enhance NLRP3 expression (Fig. 2j). Interestingly, TRIM31 deficiency greatly inhibited NLRP3 expression in LPS-primed and MG132-treated macrophages (Fig. 2j). These data indicated that MG132, as a general proteosome inhibitor, could not markedly affect NLRP3 expression in WT macrophages. However, MG132 treatment could block or reverse the effects of TRIM31 deficiency on NLRP3 expression. Taken together, these results indicated that TRIM31 could promote proteasomal degradation of NLRP3.

### TRIM31 interacts with NLRP3

**Figure 3:**
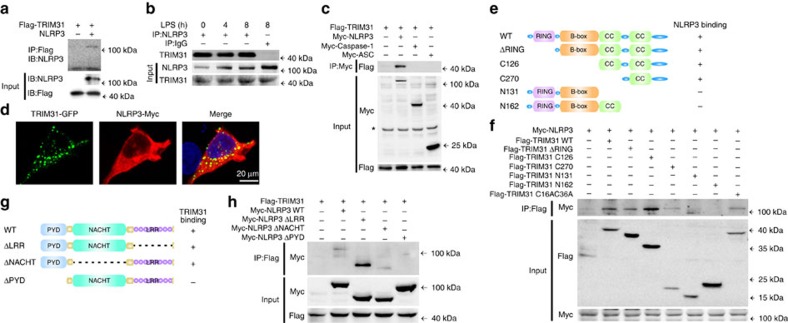
TRIM31 interacts with NLRP3. (**a**) NLRP3 and Flag-tagged TRIM31 were obtained by *in vitro* transcription and translation. Interaction between TRIM31 and NLRP3 was assayed by mixing TRIM31 and NLRP3 together followed by IP with Flag antibody and immunoblot analysis with NLRP3 antibody. (**b**) Co-immunoprecipitation of endogenous TRIM31 with endogenous NLRP3 from mouse peritoneal macrophages stimulated with LPS for indicated time periods. (**c**) HEK293T cells expressing Flag-TRIM31 and Myc-NLRP3, Myc-Caspase-1 or Myc-ASC were lysed. Co-immunoprecipitation of Flag-TRIM31 with Myc-NLRP3 from HEK293T cells. *Non-specific band. (**d**) HEK293T cells transfected with GFP-TRIM31 and Myc-NLRP3 were fixed and incubated with a secondary antibody conjugated to Alexa Fluor 568. Colocalization between TRIM31 and NLRP3 was examined by Confocal microscopy. (**e**) Schematic diagram of TRIM31 and its truncation mutants. (**f**) Flag-tagged TRIM31 or its mutants and Myc-NLRP3 were individually transfected into HEK293T cells. The cell lysates were immunoprecipitated with an anti-Flag antibody and then immunoblotted with the indicated antibodies. (**g**) Schematic diagram of TRIM31 and its truncation mutants. (**h**) Myc-tagged NLRP3 or its mutants and Flag-TRIM31 were individually transfected into HEK293T cells. The cell lysates were immunoprecipitated with an anti-Flag antibody and then immunoblotted with the indicated antibodies. Similar results were obtained in three independent experiments.

To further investigate how TRIM31 promotes NLRP3 degradation, we first examined the interaction between TRIM31 and NLRP3. *In vitro* binding assays demonstrated that TRIM31 could directly interact with NLRP3 (Fig. 3a). And then, to confirm the interaction between TRIM31 and NLRP3 *in vivo*, we assessed resting and LPS-stimulated macrophages by immunoprecipitation (IP). An association between TRIM31 and NLRP3 could be detected in both mouse peritoneal macrophages and THP-1 cells (Fig. 3b, Supplementary Fig. 3a). To investigate whether TRIM31 interacted with other NLRP3 inflammasome molecules, we expressed TRIM31 with NLRP3, caspase-1 and ASC respectively, in HEK293T cells. TRIM31 could immunoprecipitate with NLRP3, and could not immunoprecipitate with caspase-1 and ASC (Fig. 3c, Supplementary Fig. 3b). Consistently, confocal analysis also demonstrated the colocalization between TRIM31 and NLRP3 (Fig. 3d, Supplementary Fig. 3c). Taken together, these data suggested that TRIM31 could directly interact with NLRP3.

TRIM31, as a member of the TRIM family proteins, contains an N-terminal RING-finger domain, B-box domain and two C-terminal coiled-coil (CC) domains (Fig. 3e). To search for the domains of TRIM31 that is responsible for the interaction with NLRP3, a series of Flag-tagged TRIM31 truncated mutants were constructed (Fig. 3e). NLRP3 was coprecipitated with TRIM31 WT, RING domain deletion mutant (ΔRING), C126 and C270, but not with N131 and N162 (Fig. 3f). These results indicated that TRIM31 interacted with NLRP3 via its second C-terminal coiled-coil domain. Three truncated mutants of NLRP3 were also constructed (Fig. 3g). Co-IP experiments with these truncated mutants showed that NLRP3 ΔPYD lost the ability to bind TRIM31, while NLRP3 ΔLRR and ΔNACHT did not (Fig. 3h). So the PYD domain of NLRP3 is required for the NLRP3–TRIM31 interaction.

### TRIM31 promotes K48-linked polyubiquitination of NLRP3

**Figure 4:**
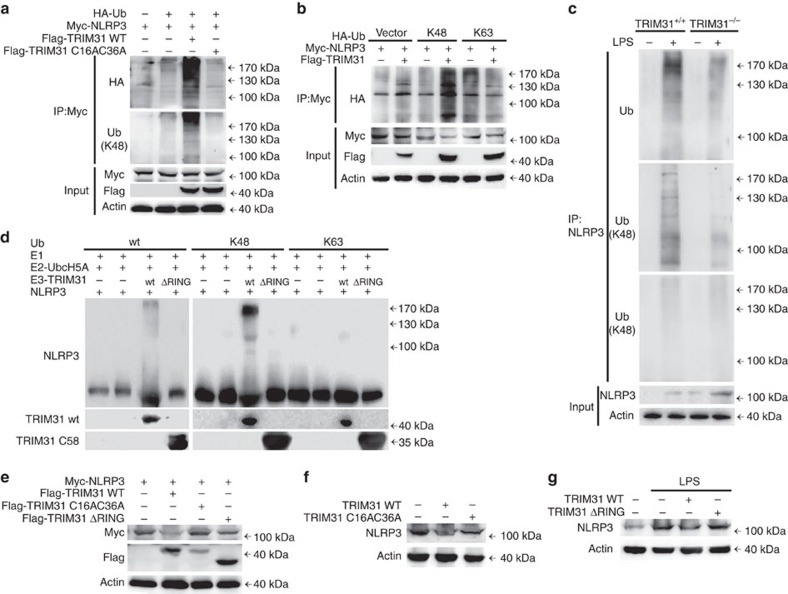
TRIM31 promotes K48-linked polyubiquitination of NLRP3. (**a**) Immunoblot analysis of lysates from HEK293T cells transfected with HA-tagged ubiquitin (HA-Ub), Myc-NLRP3 and TRIM31 WT or C16AC36A, followed by IP with anti-Myc, probed with anti-HA or K48-Ub. (**b**) Immunoblot analysis of lysates from HEK293T cells transfected with HA-tagged K48-linked ubiquitin (K48-Ub) or HA-tagged K63-linked ubiquitin (K63-Ub), Myc-NLRP3 and TRIM31, followed by IP with anti-Myc, probed with anti-HA. (**c**) Immunoblot analysis of lysates from TRIM31^+/+^ or TRIM31^−/−^ mouse peritoneal macrophages, followed by IP with anti-NLRP3, probed with anti-Ub, K48-Ub or K63-Ub. (**d**) *In vitro* ubiquitination assay was performed in the presence of Ub (wt, K48 or K63), E1, E2-UbcH5A, NLRP3 and TRIM31 (wt or ΔRING mutant). The ubiquitination of NLRP3 was examined with NLRP3 antibody. (**e**) Immunoblot analysis of extracts from HEK293T cells transfected with Myc-NLRP3 and Flag-tagged TRIM31 or its mutants. (**f**) Immunoblot analysis of extracts from THP-1 cells transfected with TRIM31 wild type (WT) or TRIM31 C16AC36A. (**g**) Immunoblot analysis of extracts from THP-1 cells transfected with TRIM31 WT or TRIM31 ΔRING then stimulated with LPS for 4 h. Similar results were obtained in three independent experiments.

Protein ubiquitination is a key step in the ubquitin–proteasome degradation pathway^28^. NLRP3 could be ubiquitinated with both K48 and K63 linkage in macrophages (Supplementary Fig. 4), and that is consistent with a previous report^18^. We have identified E3 ubiquitin ligase TRIM31 as a NLRP3-associated protein (Fig. 3) and that promoted us to investigate whether TRIM31 could mediate the ubiquitination of NLRP3. NLRP3 was cotransfected with HA-ubiquitin and WT TRIM31 into HEK293T cells. NLRP3 ubiquitination was markedly increased in the presence of TRIM31 expression plasmid (Fig. 4a). Importantly, the TRIM31 point mutation (C16AC36A) with substitution of the cysteine residue with alanine at position 16 and 36 within the RING domain, lost the ability to promote polyubiquitination of NLRP3 (Fig. 4a), indicating TRIM31 could promote the ubiquitination of NLRP3 though the N-terminal RING-finger domain. To study the forms of TRIM31-mediated NLRP3 polyubiquitination, ubiquitin mutant vectors K48 and K63, which contain arginine substitutions of all of its lysine residues except the one at position 48 and 63 respectively, were used in the transfection assays. TRIM31 promoted NLRP3 polyubiquitination could be detected in the presence of K48 plasmid, but not with K63 plasmid (Fig. 4b), indicating TRIM31 mediated K48-linked polyubiquitination of NLRP3.

Under physiological conditions, the endogenous NLRP3 was observed to be robustly ubiquitinated with both K48 and K63 linkage upon LPS stimulation (Fig. 4c). However, only K48-linked polyubiquitination of NLRP3 was almost completely abolished in TRIM31 deficient macrophages, and K63-linked ubiquitination of NLRP3 was not affected (Fig. 4c). To determine whether TRIM31 directly modified NLRP3, we reconstituted the NLRP3-ubiquitination reaction *in vitro*. We prepared recombinant NLRP3, recombinant TRIM31 (wt and ΔRING), purified E2 enzyme UbcH5A, a mixture of E1 plus ubiquitin (wt, K48 only and K63 only). We observed ubiquitination of NLRP3 only when TRIM31 wt was present (Fig. 4d). TRIM31 ΔRING lost the ability of promoting NLRP3 ubiquitination. Furthermore, we found ubiquitination of NLRP3 was mediated by K48 linkage but not by K63 linkage (Fig. 4d). Taken together, these data indicated that TRIM31 directly induced the K48-linked ubiquitination of NLRP3 through its E3 ligase activity.

K48-linked polyubiquitination leads to the degradation of target proteins through 26S proteasome^28^. TRIM31 promoted proteasomal degradation of NLRP3 (Fig. 2g). However, TRIM31 mutants ΔRING and C16AC36A with the deprivation of the E3 ubiquitin ligase activity lost the ability to promote degradation of NLRP3 (Fig. 4e–g). All together, these data demonstrated that TRIM31 mediated K48-linked ubiqutination and proteasomal degradation through its E3 ubiquitin ligase activity.

### LPS and IL-1β induce TRIM31 expression

**Figure 5:**
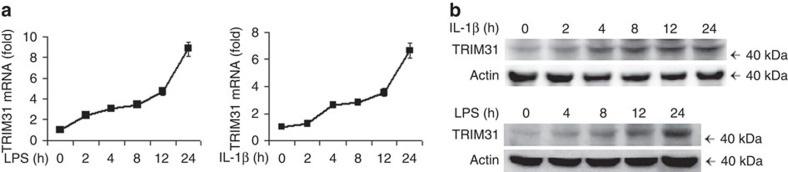
IL-1β and LPS induce TRIM31 expression. (**a**,**b**) RT-PCR analysis (**a**) or immunoblot analysis (**b**) of TRIM31 expression from mouse peritoneal macrophages stimulated with LPS or IL-1β for various times. Similar results were obtained in three independent experiments. Data are representative of three experiments (mean and s.d. of six samples in **a**).

Having established the inhibitory role of TRIM31 in NLRP3 inflammasome activation, we next examined the expression status of TRIM31 during the process of NLRP3 inflammasome activation. LPS or IL-1β stimulation greatly induced TRIM31 expression at both mRNA and protein level (Fig. 5a,b).

### TRIM31 deficiency enhances IL-1β secretion *in vivo*

**Figure 6:**
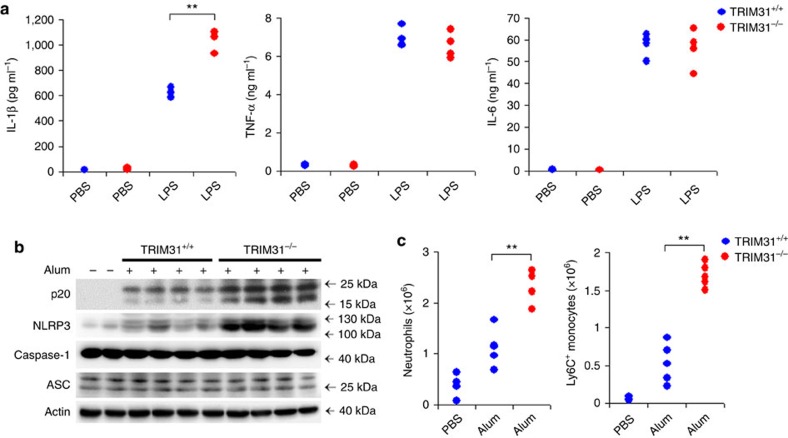
TRIM31 deficiency enhances IL-1β secretion and aggravates Alum-induced peritonitis *in vivo*. (**a**) ELISA analysis of serum levels of IL-1β, TNF-α and IL-6 from TRIM31^+/+^ or TRIM31^−/−^ mice after i.p. LPS injection. (**b**,**c**) TRIM31^+/+^ or TRIM31^−/−^ deficiency mice were i.p. injected with alum for 12 h. PECs were lysed and analysed for the expression of Caspase-1, NLRP3 and ASC by immunoblot (**b**). Absolute numbers of neutrophils or Ly6C^+^ monocytes recruited to the peritoneum were analysed by fluorescence-activated cell sorting (five mice per group) (**c**). **P<0.01. (Student's *t*-test). Data are representative of three experiments (mean and s.d. of four to five samples in **a** and **c**).

We next investigated the biological effects of TRIM31 on NLRP3 inflammasome activation *in vivo*. The induction of IL-1β by intraperitoneal (i.p.) injection of LPS is NLRP3 dependent^29^,^30^. We then examined whether TRIM31 deficiency could regulate this induction of IL-1β. TRIM31 deficiency enhanced serum concentrations of IL-1β but did not considerably increase the amount of TNF-α and IL-6 (Fig. 6a), indicating that TRIM31 could specifically attenuate NLRP3 inflammasome activation *in vivo*.

### TRIM31 deficiency aggravates alum-induced peritonitis

We further investigated the effects of TRIM31 in an IL-1-dependent mouse peritonitis model^31^. Mouse peritonitis was induced by i.p. injection of alum. Peritoneal exudate cells (PECs) were collected and alum-induced recruitment of inflammatory cells was analysed by flow cytometry. Alum challenge greatly upregulated caspase-1 cleavage in the lavage fluid and that this was significantly enhanced by TRIM31 deficiency (Fig. 6b), which was consistent with the observation *in vitro*. In addition, more NLRP3 expression was also observed in the PECs lysates from TRIM31 deficient mice (Fig. 6b). We consequently analysed alum-induced recruitment of inflammatory cells in the lavage fluid. Alum-induced recruitment of neutrophils and Ly6C^+^ monocytes was strongly enhanced in the TRIM31 deficient mice (Fig. 6c). Collectively, these findings further demonstrated that TRIM31 could inhibit NLRP3 inflammasome activity and subsequent immune cell accumulation in mouse peritonitis *in vivo*.

### TRIM31 deficiency ameliorates DSS-induced colitis

**Figure 7:**
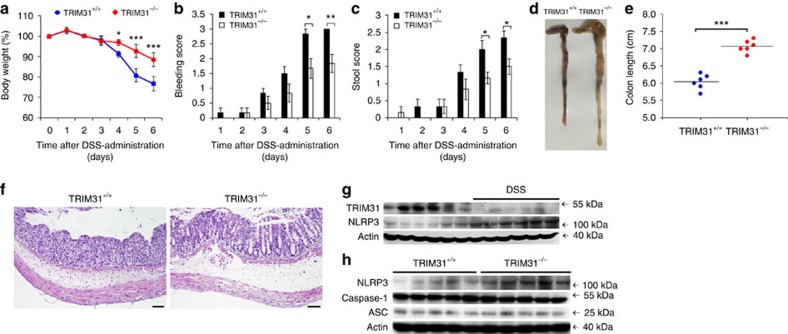
TRIM31 deficiency ameliorates DSS-induced colitis. Mice were given 3% DSS in their drinking water for 5 days, followed by regular drinking water. (**a**) Body weight, (**b**) stool consistency and (**c**) rectal bleeding score were scored daily. (**d**–**h**) Mice were killed on day 6. Macroscopic appearances (**d**) and colon lengths (**e**) of the mice were measured. Histopathological changes in colon tissue were examined by H&E staining (**f**) Scale bars, 50μm. Immunoblot analysis of lysates from colon tissue (**g**,**h**). *P<0.05, **P<0.01, ***P<0.001 (one-way analysis of variance, ANOVA).

DSS model is one of the most extensively used to investigate innate immune mechanisms of colitis^32^. NLRP3 inflammasome is critically involved in the maintenance of intestinal homeostasis and protection against colitis^33^. Besides spleen, TRIM31 expressed extremely high in gut, including small intestine and colon (Supplementary Fig. 5), indicating the functions of TRIM31 in maintaining intestinal homeostasis. We thus examined the potential roles of TRIM31 in the DDS-induced colitis model. To study the contribution of TRIM31 to the development of colitis, we first assessed the clinical features of age- and sex-matched WT and TRIM31 deficient mice after oral administration of 3% DSS in drinking water. TRIM31^−/−^ mice suffered from less body weight loss from day 4 on (Fig. 7a). Simultaneously, rectal bleeding scores and stool consistency scores of TRIM31^−/−^ mice were significantly attenuated compared with those of DSS-fed WT mice (Fig. 7b,c). To further assess the severity of colitis, colon length was measured in DSS-fed WT and TRIM31^−/−^ mice. Colons of TRIM31^−/−^ mice were longer than those of WT mice administered with DSS (Fig. 7d,e). The severity of colonic inflammation and ulceration was further evaluated by histopathological analysis using haematoxylin & eosin (H&E) staining. DSS-induced mucosal damage was characterized by crypt loss and infiltrating leucocytes. Colonic sections of DSS-fed WT mice displayed severe transmural inflammation with focal areas of extensive ulceration and necrotic lesions (Fig. 7f). Inflammatory infiltrates filled the lamina propria and submucosa in areas where the mucosa was intact and often effaced the normal architecture of the tissue (Fig. 7f). Strikingly, colonic sections of DSS-fed TRIM31^−/−^ mice exhibited less inflammatory cell infiltration and intact colonic architecture with no apparent ulceration (Fig. 7f).

To confirm the roles of TRIM31 in the model, we examined TRIM31 and NLRP3 expressions in colon tissues. In WT mice, TRIM31 constitutely expressed in colon tissues (Fig. 7g, Supplementary Fig. 5), and that maintains NLRP3 protein expression in a relatively low level (Fig. 7g). Following DSS treatment, TRIM31 was downregulated and NLRP3 protein expression was elevated (Fig. 7g). In DSS-administered TRIM31^−/−^ mice, NLRP3 protein expression in colon tissues was further enhanced (Fig. 7h). Collectively, these data indicated that TRIM31 played crucial roles in limiting NLRP3 expression and thus exacerbated DSS-induced colitis.

## Discussion

Ubiqutination is a crucial post-transcriptional modification to modulate the intensity of innate immune responses, including regulation of NLRP3 inflammasome activity. For example, K63-linked ubiquitination of ASC targets the AIM2–ASC inflammasome complex for destruction in autophagosomes^34^,^35^. The linear ubiquitin assembly complex-mediated ASC linear ubiquitination is required for NLRP3 inflammasome activation^36^. A20 restricts ubiquitination of pro-interleukin-1β protein complexes to suppress NLRP3 inflammasome activity^37^. TRIM33 ubiquitinates DHX33 and is essential for the cytosolic RNA-induced NLRP3 inflammasome activation^38^. The deubiquitination of NLRP3 by the BRCC3 complex is required for NLRP3 inflammasome activation^13^,^39^,^40^. E3 ubiquitin ligase MARCH7 promoted NLRP3 K48-linked ubiquitination and subsequent degradation in an autophagy-dependent pathway^18^. However, whether the ubiquitinated NLRP3 could be degraded via a ubiquitin–proteasome pathway is still unknown. In the present study, we found that NLRP3 could be ubiquitinated with K48 linkage in both resting and activated macrophages, and E3 ubiquitin ligase TRIM31 mediated this modification. Thus, TRIM31 promoted proteasomal degradation of NLRP3 and then inhibited the NLRP3 inflammasome activation. Collectively, several E3 ubiquitin ligases, such as TRIM31, MARCH7, DHX33 and so on, could negatively or positively regulate NLRP3 inflammasome activation at different levels and via different mechanisms. As for the regulation of NLRP3 expression, E3 ubiquitin ligase TRIM31 and MARCH7 could promote the degradation of NLRP3 via a ubiquitin–proteasome pathway and autophagy-dependent pathway, respectively. Thus, the E3 ubiquitin ligases could work together to fine tune the activation of NLRP3 inflammasome to maintain immune homeostasis. Our research identify TRIM31 as a physiological regulator of NLRP3 expression, and that will further explain the complexities of homeostatic control of innate immune signalling.

**Figure 8:**
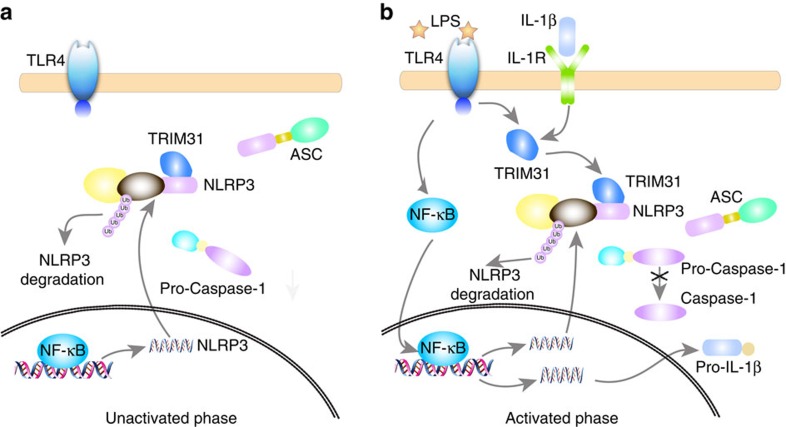
Working model for TRIM31 inhibiting NLRP3 inflammasome activation. (**a**) In resting macrophages, the constitutively expressed TRIM31 binds to NLRP3, promotes K48-linked ubiquitination and proteasomal degradation of NLRP3, and maintains its low expression. (**b**) Following NLRP3 inflammasome activation, TRIM31 expression is markedly induced by LPS and IL-1β, resulting in the inhibition of NLRP3 expression and subsequent inflammasome activation.

Based on the experimental data, we propose a model to illustrate how TRIM31 feedback negatively regulates NLRP3 inflammasome activation (Fig. 8). In resting macrophages, the constitutively expressed TRIM31-mediated proteasomal degradation of NLRP3 protein to maintain its low expression and limit the NLRP3 inflammasome assembly (Fig. 8a). Following LPS stimulation, NLRP3 expression is induced rapidly through NF-κB activation, which could facilitate NLRP3 inflammasome assembly and activation. Simultaneously, TRIM31 expression is induced by LPS stimulation and IL-1β secretion. TRIM31 then directly bound to NLRP3 and promoted its K48-linked ubiquitination, leading to its proteasomal degradation (Fig. 8b). Thus, the activation of NLRP3 inflammasome was limited in a proper intensity and time course to avoid detrimental effects.

Improper NLRP3 inflammasome activation has been linked to a variety of diseases^5^, such as infectious diseases, autoinflammatory and autoimmune diseases. NLRP3 inflammasome activity is critical for host response to microbial pathogens. Excessive NLRP3 inflammasome activation plays crucial causative or contributing roles in the initial of autoinflammatory and autoimmune diseases^5^. However, optimal NLRP3 inflammasome activation is beneficial for some inflammatory disorders, such as inflammatory bowel diseases^33^. Thus, a better understanding of the balance between beneficial and detrimental NLRP3 inflammasome activation is needed.

In the present study, we found that TRIM31 constitutely expressed in macrophages and negatively regulated NLRP3 inflammasome activation to prevent unwanted activation in both resting and activation state. *In vivo* experiments also confirmed the beneficial roles of TRIM31 in the alum-induced peritonitis, an NLRP3 dependent acute inflammatory model. Furthermore, TRIM31 also constitutively expressed in gut, limited NLRP3 expression in a relatively low level and thus possessed potential functions in maintaining intestinal homeostasis. However, TRIM31^+/+^ mice were more susceptible to acute DSS-induced colitis, compared with TRIM31^−/−^ mice. Recent evidence has indicated that NLRP3 confers protection against acute colitis^33^,^41^,^42^. TRIM31 expression was downregulated in the DSS-induced colitis model, and NLRP3 expression was upregulated accordingly. Thus, NLRP3 could exert its protection roles when colitis was initiated. In TRIM31^−/−^ mice, NLRP3 expression was further increased in the colon of DSS-induced colitis model. So, TRIM31 deficiency ameliorates DSS-induced colitis.

In summary, we identified TRIM31 as a feedback suppressor of NLRP3 inflammasome activity. Because of the conserved biological function of NLRP3 inflammasome in multiple physiological processes, fine-tuning of its activity is critical for the disease resistance and maintenance of immune homeostasis. Our research provides an explanation on the limitation of NLRP3 inflammasome activation under physiological conditions. Furthermore, TRIM31 may provide a useful therapeutic target for the intervention of diseases with improper inflammasome activation, such as autoinflammatory diseases, colitis, obesity and diabetes.

## Methods

### Mice

TRIM31^−/−^ mice on a C57BL/6J background were generated by Cyagen Biosciences Inc. (Guangzhou, China) using TALEN technology. A pair of TALEN constructs for TRIM31 knockout (KO) were cloned into a mammalian expression vector pCMV-TALEN and capped, polyA-tailed mRNA for injection were produced using the Ambion mMessage mMachine kit. The KO mice were produced by microinjecting TALEN mRNAs into fertilized eggs from C57BL/6 strain. The KO alleles have been sequence validated. Three kinds of KO mice were generated and named as KO1, KO2 and KO3. KO1 has one missing base pairs (C, 97 of the ORF), KO2 has one missing base pairs (G, 94 of the ORF) and KO3 has five missing base pairs (GGGCA, 94-98 of the ORF). All the three KOs caused a frame shift. The mRNA transcribed from targeted allele with frame shift undergoes nonsense-mediated decay. KO1 mice were used in most experiments if no special noted. The mice were backcrossed with C57BL/6 mice for more than six generations. C57BL/6 mice were obtained from Vital River Laboratory Animal Technology Co. (Beijing, China). All animal experiments were undertaken in accordance with the National Institute of Health Guide for the Care and Use of Laboratory Animals, with the approval of the Scientific Investigation Board of Medical School of Shandong University (Jinan, Shandong Province, China).

### Reagents

ATP, MG132, chloroquine, cycloheximide, phorbol myristate acetate, LPS (*Escherichia coli*, 055:B5), peptidoglycan (PGN), poly(I:C), 3-MA, anti-TRIM31, anti-Myc and anti-Flag were from Sigma-Aldrich (St Louis, MO); Dextran sulfate sodium (DSS, 36-50 kDa) was bought from MP Biomeicals (Aurora, OH). Imject Alum was from Thermo Scientific; IL-1β and TNF-α were from PeproTech (Rocky Hill, NJ); Flagellin and poly(dA:dT) were from Invivogen (San Diego, CA). Nigericin, anti-caspase-1 p45&p20, anti-NLRP3 and anti-ASC were from AdipoGen; anti-hemagglutinin (HA), anti-Ub, anti-β-actin, protein G agarose used for IP and horseradish peroxidase-conjugated secondary antibodies were from Santa Cruz Biotechnology (Santa Cruz, CA); anti-IL-1β p31&p17, anti-p-JNK, anti-p-p38, anti-p-ERK, anti-JNK, anti-p38, anti-ERK1/2, anti-p-IκBα, anti-K48-ub and anti-K63-ub were from Cell Signaling Technology (Beverly, MA); anti-AIM2 and anti-NLRC4 were from Abcam (Cambridge, MA).

### Cell culture

To obtain mouse primary peritoneal macrophages, C57BL/6J mice (female, 4–6 weeks old) were injected intraperitoneally with 3% Brewer's thioglycollate broth. Three days later, PEC were harvested and incubated. Two hours later, nonadherent cells were removed and the adherent monolayer cells were used as peritoneal macrophages^14^,^40^,^43^. Human THP-1 and human embryonic kidney (HEK293T) cells were obtained from American Type Culture Collection (Manassas, VA). Phorbol myristate acetate-activated THP-1 cells were used as human macrophages. The cells were cultured at 37 °C under 5% CO_2_ in DMEM supplemented with 10% FCS (Invitrogen-Gibco), 100 U ml^−1^ penicillin, and 100 μg ml^−1^ streptomycin. The concentration of agonists or stimuli were used as below: LPS 100 ng ml^−1^ for mouse primary peritoneal macrophages, LPS 1 μg ml^−1^ for THP-1 cells, ATP 5 mM, nigericin 50 μM and alum 200 μg ml^−1^, IL-1β 10 ng ml^−1^, poly(I:C) 10 μg ml^−1^, TNF-α 200 ng ml^−1^ and PGN 5 μg ml^−1^. Poly(dA:dT) and flagellin were transfected into macrophages with the final concentration as below: poly(dA:dT) 200 ng ml^−1^ and flagellin 200 ng ml^−1^.

### Plasmids construction and transfection

TRIM31 expression plasmid was constructed by PCR-based amplification of cDNA from THP-1 cells, and then cloned into the pFLAG-CMV-2 eukaryotic expression vector (Sigma-Aldrich). Caspase-1 expression plasmid was constructed by PCR-based amplification of cDNA from THP-1 cells, and then cloned into the pCMV-Myc eukaryotic expression vector (Beyotime, China). Myc-NLRP3 and Myc-ASC plasmids were provided by Dr John C. Reed (Sanford-Burnham Medical Research Institute, La Jolla, CA). Expression vectors for HA-Ub WT, mutant K48 and mutant K63 were from Dr Hui Xiao (Institut Pasteur of Shanghai, CAS, Shanghai, China). MyD88 plasmid was gifts from Dr Xuetao Cao (Second Military Medical University, Shanghai, China). NF-κB reporter plasmid was purchased from Stratagene. Truncated mutants of TRIM31 or NLRP3 were generated using the KOD-Plus-Mutagenesis kit (Toyobo, Osaka, Japan). All constructs were confirmed by DNA sequencing. Plasmids were transiently transfected into HEK293T cells or THP-1 cells with jetPRIME reagents (Polyplus) according to the manufacturer's instructions.

### RNA interference assay

siRNAs were synthesized as following: murine TRIM31: 5′-GCUCACUAAAUCCUUGAAA-3′, human TRIM31: 5′-GGACCACAAAUCCCAUAAU-3′ and negative control: 5′-UUCUCCGAACGUGUCACGU-3′. These siRNA duplexes were transfected into mouse peritoneal macrophages or THP-1 cells using INTERFERin reagents (PolyPlus) according to the manufacturer's instructions.

### ELISA

The concentrations of mouse IL-1β, mouse TNF-α and mouse IL-6 were measured using ELISA kits (Dakewe Biotech Company Ltd., Shenzhen, China) according to the manufacturer's instruction.

### RNA quantitation

Total RNA was extracted with TRIzol reagent according to the manufacturer's instructions (Invitrogen). A LightCycler (ABI PRISM 7000) and a SYBR RT-PCR kit (Takara) were used for quantitative real-time RT-PCR analysis. Specific primers used for RT-PCR assays were the sequences of primers used for RT-PCR were 5′-TGGATGGGTTTGCTGGGAT-3′, 5′-CTGCGTGTAGCGACTGTTGAG-3′ for NLRP3; 5′-ACCTTCCAGGATGAGGACATGA-3′, 5′-AACGTCACACACCAGCAGGTTA-3′ for IL-1β; 5′-CCAGAGTCAAACCGTGAGCG-3′, 5′-GGCAACTTGGAGCCCGAA-3′ for TRIM31; 5′-GCCACCACGCTCTTCTGTCT-3′, 5′-TGAGGGTCTGGGCCATAGAAC-3′ for TNF-α; and 5′-TGTTACCAACTGGGACGACA′3, 5′-CTGGGTCATCTTTTCACGGT-3′ for β-actin. Data are normalized to β-actin expression in each sample.

### Immunoprecipitation and immunoblot analysis

For IP, whole-cell extracts were lysed in IP buffer containing 1.0% (vol/vol) Nonidet P 40, 50 mM Tris–HCl, pH 7.4, 50 mM EDTA, 150 mM NaCl, and a protease inhibitor ‘cocktail' (Merck). After centrifugation for 10 min at 14,000 *g*, supernatants were collected and incubated with protein G Plus-Agarose Immunoprecipitation reagent together with specific antibody. After 6 h of incubation, beads were washed five times with IP buffer. Immunoprecipitates were eluted by boiling with 1% (wt/vol) SDS sample buffer. For immunoblot analysis, cells were lysed with M-PER Protein Extraction Reagent (Pierce, Rockford, IL) supplemented with a protease inhibitor ‘cocktail', then protein concentrations in the extracts were measured with a bicinchoninic acid assay (Pierce, Rockford, IL). Equal amounts of extracts were separated by SDS–polyacrylamide gel electrophoresis (PAGE), and then were transferred onto nitrocellulose membranes for immunoblot analysis. Cell culture supernatants were harvested and concentrated for immunoblot with Amicon Ultra 10 K from Millipore. The concentration of primary antibodies used for immunoblot was 1 μg ml^−1^. The secondary antibodies were used 1 in 5,000. Uncropped scans of immunoblots are provided as Supplementary Figs 6–8.

### *In vitro* binding and ubiquitination assay

NLRP3, TRIM31 WT and ΔRING mutant proteins were expressed with a TNT Quick Coupled Transcription/Translation System (Promega) according to the instructions of the manufacturer^22^. Binding assays were performed by mixing Flag-tagged TRIM31 and NLRP3 together, followed by IP with Flag antibody and WB with NLRP3 antibody. Ubiquitination was analysed with an ubiquitination kit (Boston Biochem) following protocols recommended by the manufacturer.

### Luciferase assay

Luciferase activities were measured with Dual-Luciferase Reporter Assay System (Promega) on a microplate luminometer (Centro LB 960; Berthold, Wildbad, Germany) according to the manufacturer's instructions. Data are normalized for transfection efficiency by subtracting Firefly luciferase activity with that of Renilla luciferase.

### Immunofluorescence staining and confocal analysis

HEK293T cells transiently transfected with plasmids encoding Myc-NLRP3 and GFP-TRIM31 were cultured for 24 h. Myc-tagged NLRP3 was detected directly following fixation and washing. Anti-Myc Ab was used at 1:1,000 dilutions in the blocking solution. The cells were then incubated with Alexa Fluor 568-conjugated secondary Ab (Molecular Probes, Invitrogen) diluted 1:1,000 in blocking solution. Nuclei were stained with DAPI (4′, 6′-diamidino-2-phenylindole hydrochloride; Molecular Probes, Invitrogen). Cells were examined with confocal laser microscopy (LSM780, Carl Zeiss, Oberkochen, Germany).

### *In vivo* LPS challenge

WT or TRIM31 deficiency mice (females, 6 weeks old) were i.p. injected with 10 mg kg^−1^ LPS or PBS^30^. After 2 h mice were killed, and serum levels of IL-1β, TNF-α and IL-6 were measured by ELISA.

### *In vivo* peritonitis

WT or TRIM31 deficiency mice (females, 6 weeks old) were i.p. injected with 700 μg alum for 12 h. Peritoneal cavities were washed with 6 ml of PBS. The peritoneal fluids were harvested and concentrated for ELISA analysis with Amicon Ultra 10 K from Millipore. PECs were analysed by FACS^14^,^31^.

### DSS-induced colitis

Colitis was induced in WT or TRIM31 deficiency C57BL/6 mice (females, 6 weeks old) with 3% (wt/vol) DSS dissolved in drinking water for 5 days, followed by regular drinking water. Mice were monitored for body weight, stool consistency and the presence of occult blood every day. Scoring for stool consistency and occult blood was done as described previously^32^,^33^. In brief, stool scores were determined as follows: 0, well-formed pellets; 1, semiformed stools that did not adhere to the anus; 2, semiformed stools that adhered to the anus; 3, liquid stools that adhered to the anus. Bleeding scores were determined as follows: 0, no blood as tested with hemoccult (Beckman Coulter); 1, positive hemoccult; 2, blood traces in stool visible; 3, gross rectal bleeding. After day 6, the entire colon was excised to measure the length of the colon and the weight of caecum. Then ∼0.5 cm of mice colon tissues close to the rectum was collected for histological analysis and protein extraction. For histological analysis, formalin-fixed and paraffin-embedded segments of colon tissue were sectioned at 4 mm in thickness, and the sections were stained with hematoxylin and eosin (H&E). TRIM31, NLRP3, Caspase-1 and ASC expression were examined with immunoblot analysis.

### Statistical analysis

All experiments were independently performed three times. Data are presented as means±s.d. of three or four experiments. Analysis was performed using a Student's *t*-test or One-way analysis of variance (ANOVA). Values of *P*<0.05 were considered to be statistically significant.

### Data availability

The data that support this study are available within the article and its Supplementary Information files or available from the authors upon request.

## Additional information

**How to cite this article**: Song, H. *et al*. The E3 ubiquitin ligase TRIM31 attenuates NLRP3 inflammasome activation by promoting proteasomal degradation of NLRP3. *Nat. Commun.* **7**, 13727 doi: 10.1038/ncomms13727 (2016).

**Publisher's note:** Springer Nature remains neutral with regard to jurisdictional claims in published maps and institutional affiliations.

## Supplementary Material

Supplementary Information Supplementary Figures
