# Supplementary material for: The E3 ubiquitin ligase TRIM31 attenuates NLRP3 inflammasome activation by promoting proteasomal degradation of NLRP3
Source: Nat Commun. 2016 Dec 8;7:13727. doi: 10.1038/ncomms13727 (PMC5155141; doi:10.1038/ncomms13727)

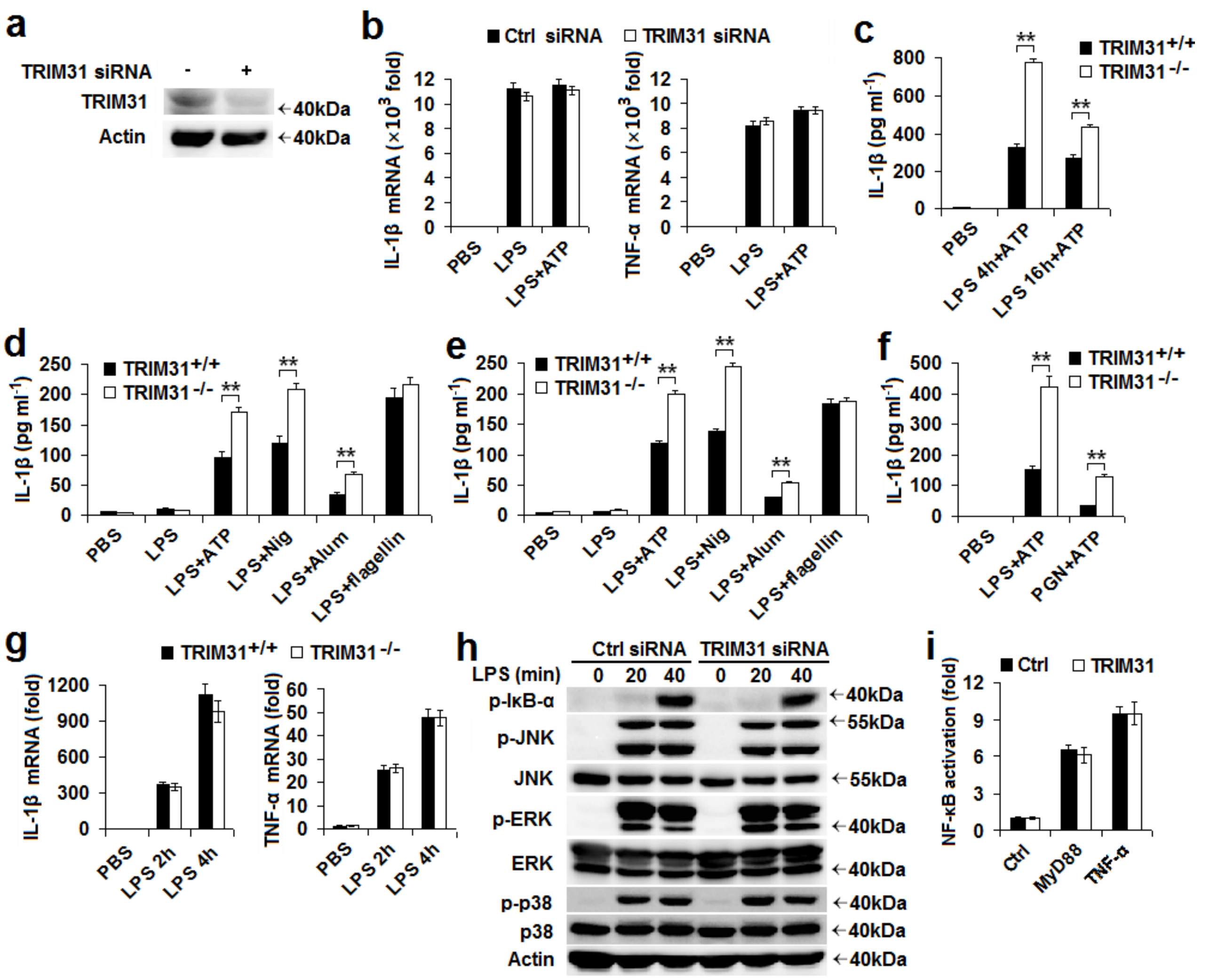

Supplementary Figure 1. (**a**) Western blot analysis of TRIM31 expression in mouse peritoneal macrophages transfected with scrambled control siRNA or TRIM31 siRNA for 36 h. (**b**) RT-PCR analysis of IL-1β and TNF-α mRNA expression in mouse peritoneal macrophages transfected with siRNA as indicated and stimulated as indicated. (**c**) ELISA of IL-1β in supernatants of mouse peritoneal macrophages from TRIM31$^{+/+}$ or TRIM31$^{-/-}$ mice, primed with LPS for indicated time periods, and followed by stimulation with ATP (5 mM) for 30 min. (**d**) ELISA of IL-1β in supernatants of mouse peritoneal macrophages from TRIM31$^{+/+}$ or TRIM31$^{-/-}$ (KO2) mice, primed with LPS for 8 h, and followed by stimulation with ATP, Nig., poly(dA:dT) or flagellin for 30 min. (**e**) ELISA of IL-1β in supernatants of mouse peritoneal macrophages from TRIM31$^{+/+}$ or TRIM31$^{-/-}$ (KO3) mice, treated as in (**d**). (**f**) ELISA of IL-1β in supernatants of mouse peritoneal macrophages from TRIM31$^{+/+}$ or TRIM31$^{-/-}$ mice, primed with LPS or PGN for 8 h, and followed

by stimulation with ATP for 30 min. (**g**) RT-PCR analysis of IL-1β and TNF-α of mouse peritoneal macrophages from TRIM31$^{+/+}$ or TRIM31$^{-/-}$ mice stimulated with LPS for various time. (**h**) Immunoblot analysis of lysates from mouse peritoneal macrophages silenced of TRIM31 stimulated with LPS for various times. (**i**) Luciferase activity of HEK293 cells transfected with an NF-κB luciferase reporter and TRIM31 plasmid, together with vector for MyD88 or stimulated with TNF-α. **, p<0.01. (Student's *t*-test). Data are representative of three experiments (mean and s.d. of six samples).

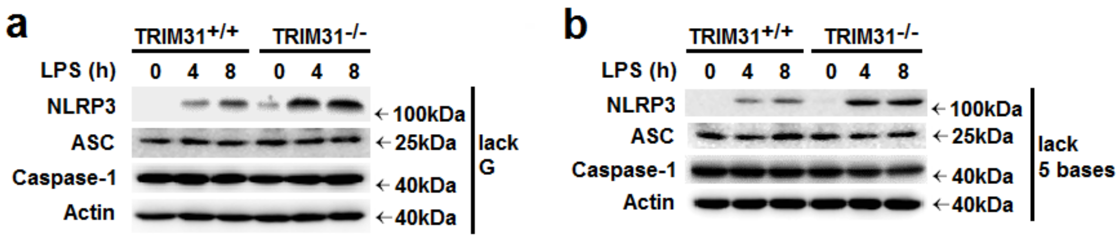

Supplementary Figure 2. (**a**) Immunoblot analysis of extracts from TRIM31$^{+/+}$ or TRIM31$^{-/-}$ (KO2) mouse peritoneal macrophages, then stimulated for various times with LPS. (**b**) Immunoblot analysis of extracts from TRIM31$^{+/+}$ or TRIM31$^{-/-}$ (KO3) mouse peritoneal macrophages, then stimulated for various times with LPS.

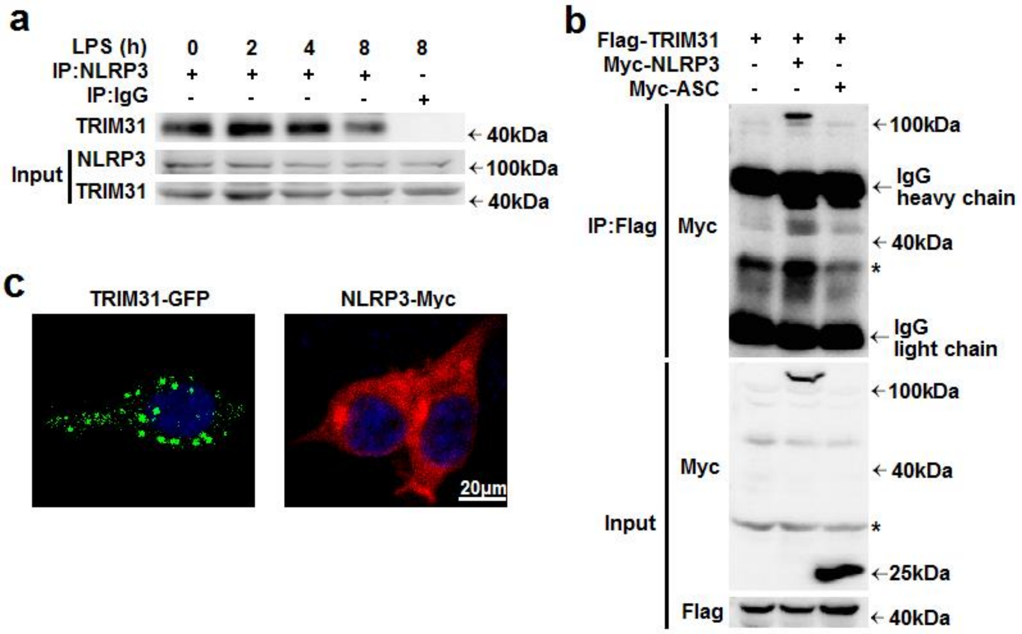

Supplementary Figure 3. (**a**) Coimmunoprecipitation of endogenous TRIM31 with endogenous NLRP3 from THP-1 cells stimulated with LPS for indicated time periods. (**b**) HEK293 cells expressing Flag-TRIM31 and Myc-NLRP3 or Myc-ASC were lysed. Coimmunoprecipitation of Myc-NLRP3 with Flag-TRIM31 from HEK293 cells. *, non-specific band. (c) HEK293T cells transfected with GFP-TRIM31 or Myc-NLRP3 were fixed and incubated with a secondary antibody conjugated to Alexa Fluor 568. TRIM31 or NLRP3 distribution was examined by Confocal microscopy.

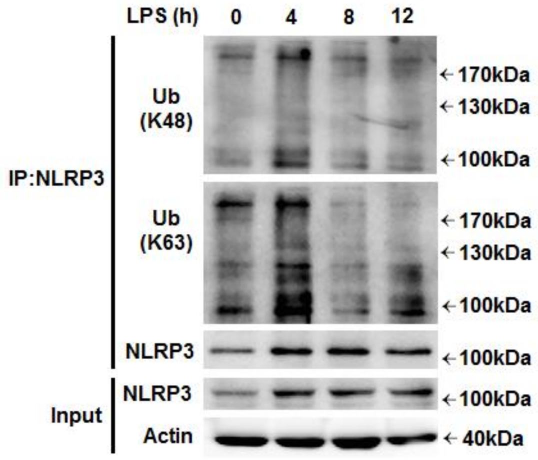

Supplementary Figure 4. Immunoblot analysis of lysates from THP-1 stimulated for various times with LPS, followed by immunoprecipitation with anti-NLRP3, probed with K48-ub or K63-ub.

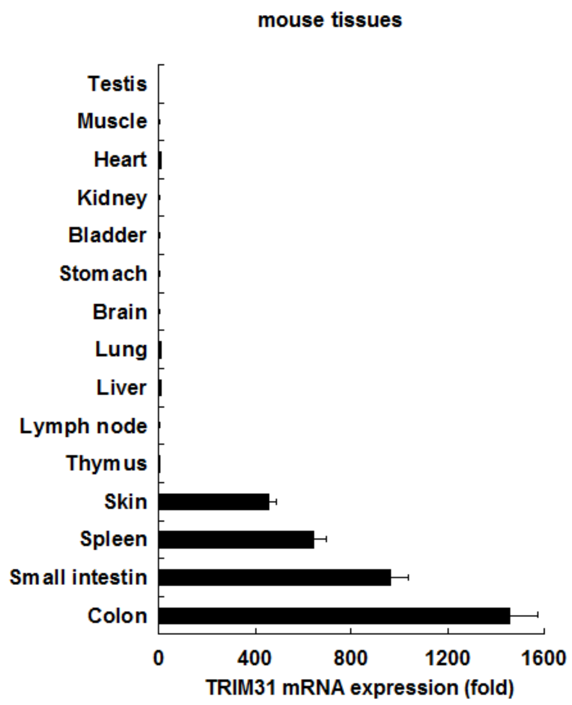

Supplementary Figure 5. RT-PCR analysis TRIM31 mRNA expression in various mouse tissues. Data are representative of three experiments (mean and s.d. of three samples).

Supplementary Figure 6. Scans of the full films used to generate Western blot data for figure 1-3.

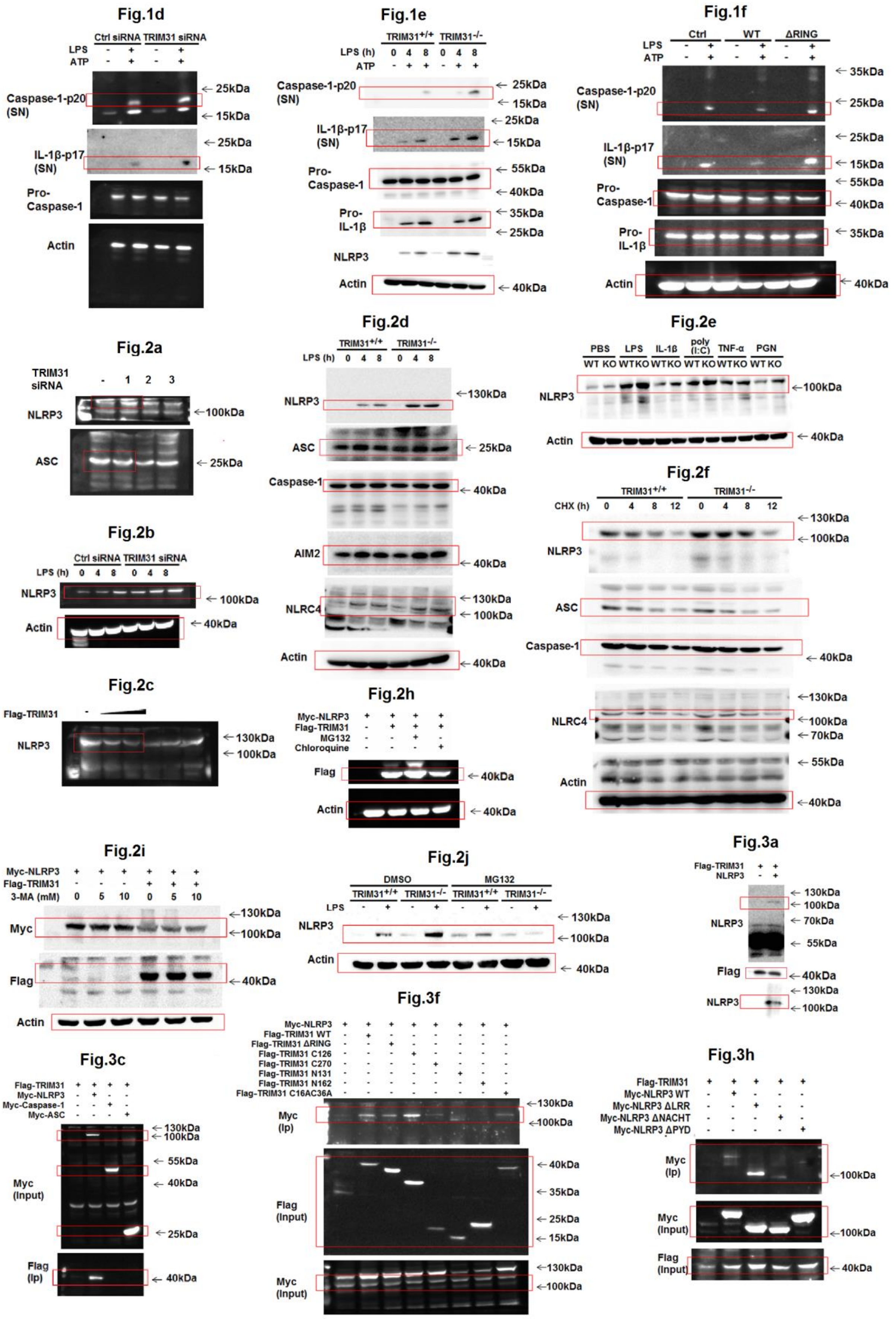

Supplementary Figure 7. Scans of the full films used to generate Western blot data for figure 4-7.

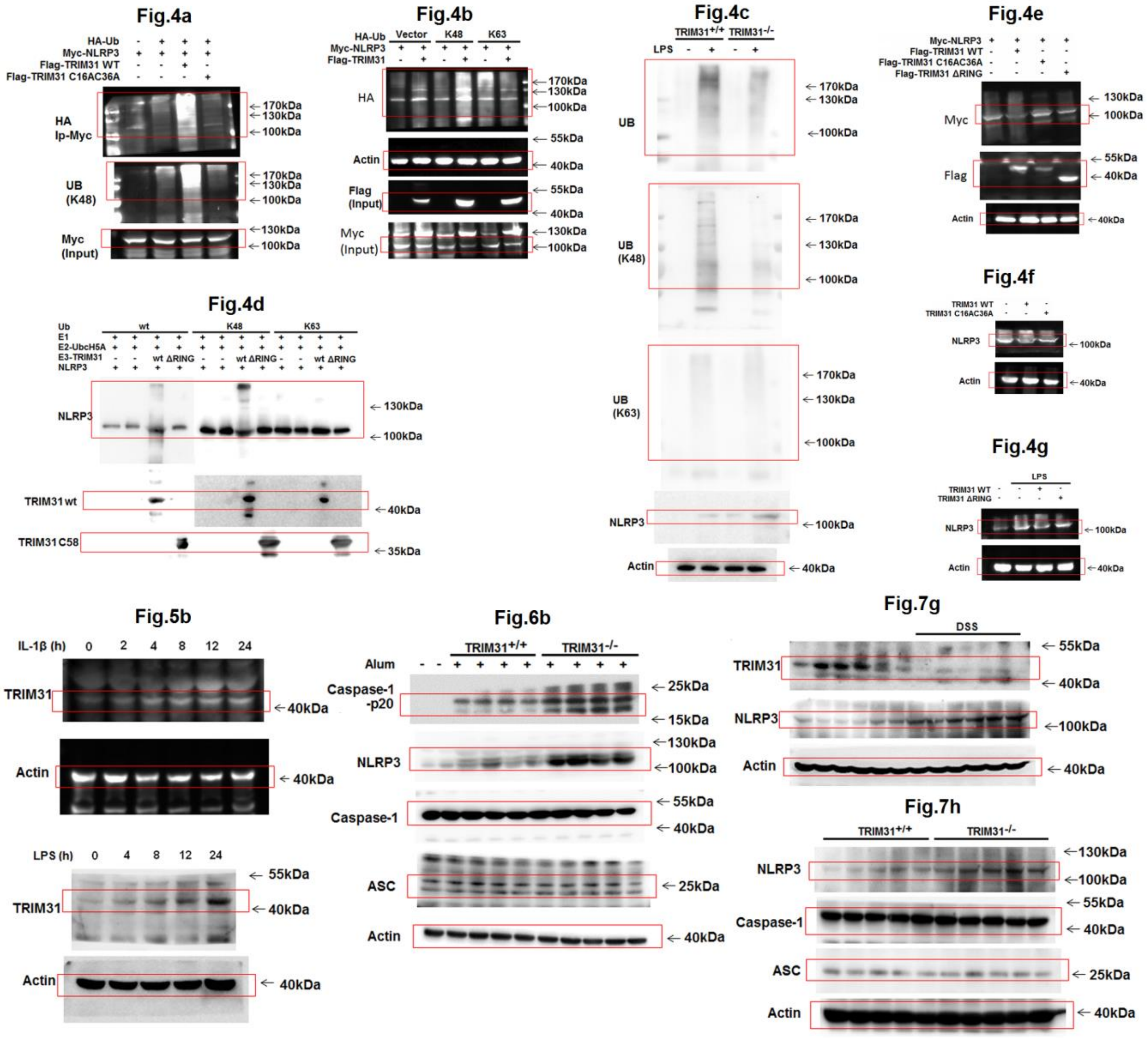

Supplementary Figure 8. Scans of the full films used to generate Western blot data for supplementary figures.

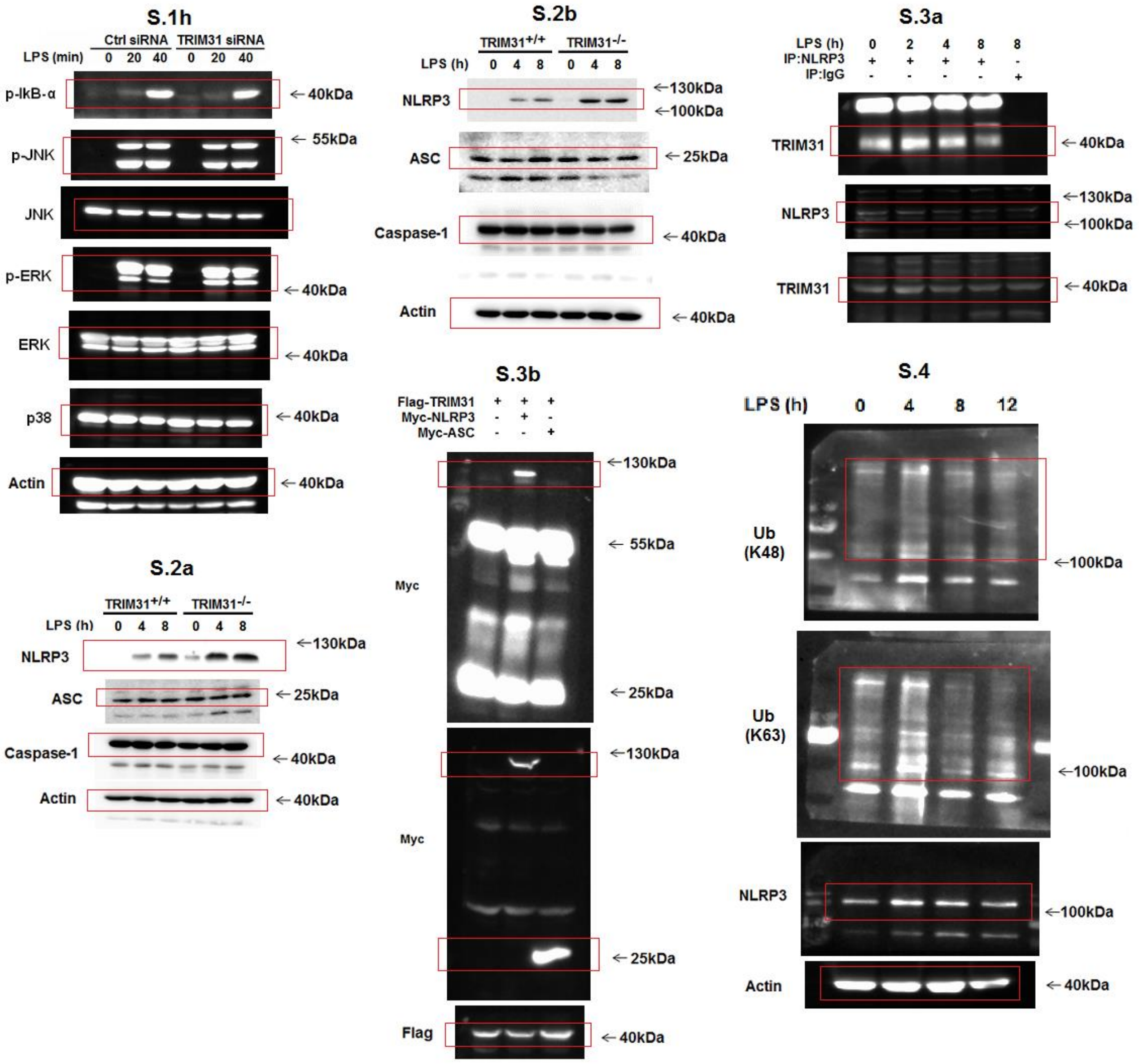

Supplement: Supplementary Information — Supplementary Figures [file ncomms13727-s1.pdf]
